# Effects of Servicescapes on Interaction Quality, Service Quality, and Behavioral Intention in a Healthcare Setting

**DOI:** 10.3390/healthcare11182498

**Published:** 2023-09-08

**Authors:** Sohyun An, Pyoungsoo Lee, Choong Ho Shin

**Affiliations:** 1 Graduate School of Service Management, Kyonggi University, Seoul 03753, Republic of Korea; 2 Division of Business Administration, Kyonggi University, Suwon 16227, Republic of Korea

**Keywords:** healthcare, servicescape, interaction quality, service quality, behavioral intention

## Abstract

This study develops a conceptual framework that encompasses servicescapes and customer perceptions and behaviors, and conducts an empirical investigation of healthcare service facilities. Structural equation modeling is performed using a sample of 271 patients who received treatment within one year at hospitals and clinics located in the metropolitan area of Seoul, South Korea. The results of the empirical analysis show that service quality improvements and patient revisits to healthcare facilities can be induced through servicescape improvements and interaction quality. These results make theoretical contributions to the service management literature and have practical implications for the operations of healthcare facilities.

## 1. Introduction

Servicescapes, defined as the physical surroundings in which customer experiences are created, are regarded as a key driver of customers’ overall perceptions of a service and their related behavior [1,2]. A servicescape provides customers with a first impression of the service, which inevitably has a significant impact on their overall evaluation of that service. Many studies have consistently provided empirical evidence on how servicescapes affect customers’ perceptions and behavioral responses. Customers’ overall evaluation of services has been studied, mainly based on service quality. However, the relationship between servicescapes and service quality has received relatively little attention. Specifically, these two concepts were not analyzed together using a structurally valid research model because of the overlap between them [3].

Traditionally, servicescapes include ambient conditions such as temperature, noise, and odor, as well as tangible elements such as furniture, signage, decorations, brochures, and other communication materials [1,3,4]. In other words, the physical environment is a concept that encompasses tangible and intangible elements. Many previous studies on service quality have also used tangible factors to measure it. Typically, SERVQUAL, the most widely used service quality measure, also evaluates service quality by including tangible factors. Additionally, interpersonal interactions, which are considered a vital factor in service, combine with these two concepts: tangibles and intangibles. For this reason, quality assessment through service environments may not have been dealt with in detail due to incompatible conceptualizations. Therefore, a few recent studies have made efforts to structurally analyze the impact of servicescapes on service quality and/or customer behavior [5,6,7]. Along this line of research, we investigate the relationship between servicescapes and customers’ perceptions of services using tangible and interactive aspects from conceptualizations used in previous studies. A customer’s perception is captured by the interaction quality and overall service quality, while the customer’s behavior is set as a revisit intention to present a conceptual framework encompassing the servicescape, customer perception, and behavior.

Services is a very broad term and is used as a concept encompassing that provided in fields such as education, hospitality, transportation, entertainment, healthcare, and so on. The conceptual model we propose is illustrated in the healthcare service, as healthcare delivery differs from other service industries. Although generally applicable theories on service evaluation and customer perception have been studied for a long time, it is difficult to apply these theories uniformly to all service industries. As healthcare services involve specialized technologies, personnel, and equipment, patients have difficulty in determining, measuring, and evaluating the technical nature of healthcare services [8]. As a result, the surroundings of facilities play an important role in determining the overall service quality [9,10] and also influence the nature of the relationship between staff and patients [8,11,12]. In addition, the influence of the mentioned environmental factors on service quality or relationship characteristics may show different characteristics from other services. Therefore, it is important to determine the components that can help patients achieve better outcomes [6]. 

The main purpose of this study is to increase the level of theoretical understanding of the role of servicescapes in determining patients’ perceptions of interpersonal interactions and overall service quality. Additionally, the theoretical framework is extended to patients’ intentions to revisit and practical implications are drawn by focusing on servicescape management in healthcare organizations.

## 2. Theoretical Background

### 2.1. Servicescapes

Even before the term servicescape appeared, scholars recognized the importance of the physical environment and tried to analyze its influence on consumers’ emotions or behavioral responses. For instance, Kotler [13] used the word “atmospherics” to define the physical factors that affect consumers, suggesting that they should be designed and controlled to generate certain emotions by providing consumers with a significant purchasing signal or reinforcement. The stimulus–organism–response (S–O–R) framework [14], which was proposed around the same time, has also been widely used to investigate the influence of the environment on consumers’ emotions and behaviors. 

Baker [15] argued that the physical environment in the field of service management can stimulate purchases by creating a positive atmosphere. Subsequently, Bitner [1] coined the term “servicescape” as a compound of service and landscape and defined it as a “built environment”, that is, an artificial physical environment as opposed to the natural or social environment. She also explained that servicescapes should be able to support interactions by simultaneously satisfying the needs and preferences of employees and customers. Although some differences in servicescape dimensions have been suggested in previous studies, most of them use a multidimensional perspective. Baker [15] divided the dimensions of servicescapes into ambient, design, and social factors. Ambient factors include lighting in the service environment and an appropriate room temperature, which can be perceived indirectly by consumers. Design factors are elements that can be seen and include the exterior, color, stability, and functional aspects of service facilities. Social factors include the recognition that human interactions and stimuli influence customers’ experiences. Bitner [1] suggested that the dimensions of the servicescape are “ambient conditions”, “spatial layout and functionality”, and “signs, symbols, and artifacts”. Ambient conditions can be defined similarly to Baker [15], being background characteristics of the physical environment that can influence consumers’ feelings, reactions, and emotions. Spatial layout and functionality refer to the suitability of consumables or furniture arrangement for service facilities. Signs, symbols, and artifacts refer to various signals, building guide maps, company logos, symbolic objects, and ornaments that facilitate communication between consumers and employees. 

In this section, the discussion focuses on the two subjects in servicescape research. The first one concerns the interaction between service employees and consumers. Baker [15] and Bitner [1], as the dominant typologies in this research stream, consider the factors related to the relationships between employees and consumers but differ in their approach as follows. Baker [15] explained these contents under a social dimension, whereas Bitner [1] introduced them into the overall servicescape concept in a somewhat indirect way rather than constructing and explaining them as an independent dimension. Based on Baker’s [15] cognitive system, some scholars treated social factors as the characteristics of employees or customers in the service environment [16,17]. However, social factors should be recognized as interactions that occur in the environment and not as environmental factors themselves. According to Bitner [1], servicescapes should be able to support interactions by simultaneously satisfying the needs and preferences of employees and customers; thus, it makes sense to analyze interactions as a result of environmental stimuli. Hutton and Richardson [9] supported this discourse by examining the effects of the factors in the physical healthcare environment on patient (customer) and staff (employee) behaviors and their outcomes.

The second argument is the controllability of servicescapes. Rosenbaum and Massiah [16] extended the view that uncontrollable factors such as the natural environment should also be included in servicescapes. This assertion emphasizes that factors such as the healing environment outside a medical service facility also needs to be analyzed, as this is conducive to the psychological stability of patients [18]. Further, drawing from Bitner [1], Wakefield and Blodgette [4] conceptualized the servicescape of leisure services by excluding uncontrollable ambient factors in the outdoor environment and adding a controllable dimension, such as cleanliness. However, unless research is intended to determine the location of a service facility, defining servicescapes in terms of uncontrollable factors such as the natural environment is rather limited. Hooper et al. [5] argued that, although there are various perspectives of the servicescape dimension, existing studies typically consist of elements such as ambient conditions, design, space and layout, equipment, hygiene, and cleanliness. Despite the relative ease with which this dimension can be controlled, Hoffman et al. [19] reported that cleanliness issues are the most frequently cited servicescape failures by consumers. More recently, Pai and Chary [20] conceptualized and analyzed healthscapes based on visual appeal and layout, amenity, and cleanliness and hygiene, and showed that cleanliness and hygiene are the most important aspects of a healthscape. This is likely because maintaining a clean and hygienic environment should be one of the cheapest and easiest servicescape dimensions to control. In medical facilities, hygiene is a factor directly related to patient health; therefore, it is necessary to analyze it as a servicescape. Nevertheless, hygiene has received relatively little attention in servicescape research. It is thus necessary to analyze hygiene together with other elements of the servicescape that are traditionally considered.

### 2.2. Interaction Quality

In service delivery, the service organization and consumers must form a relationship through interaction so that both can achieve positive results [21,22]. In relational marketing, employee–customer interactions have been treated as a vital marketing element [23,24,25]. In general, the relationship marketing perspective emphasizes satisfaction, trust, empathy, confidence, cooperation, interdependence, and social exchange as factors important for forming interpersonal relationships. Consumers can fulfil the role of co-producers through interactions with service providers in the service process, leading to continuous economic exchanges. This issue has been addressed in many empirical studies on the importance of interpersonal relationships in various service settings [26,27,28,29].

King and Garey [30] pointed out that a lack of interest in relational quality occurred in the interactions between customers and service staff, as a prerequisite for customer satisfaction. Since then, several studies have empirically shown that interpersonal interactions have a significant impact on customers’ perceptions of service quality [31,32,33]. Mattila and Enz [34] emphasized that consumers’ evaluations of the service process have a high correlation with the emotions that they feel while interacting with employees during the service delivery process. Jamal and Naser [35] and Ekinci and Dawes [36] showed that relational (interaction) quality directly affects customer satisfaction. Hooper et al. [5] explained that interpersonal relationships are created through interaction, and argued that interaction quality may differ from the overall service quality. It can thus be inferred that interaction quality is an important service quality factor between consumers and employees and a medium that can help and satisfy the needs of consumers.

The healthcare literature continues to emphasize the importance of interaction quality. For instance, Thom et al. [37] emphasized the importance of interaction in quality-of-care evaluations and found that the more the patient trusts the medical staff, the more positive is the intention to revisit a medical institution. Chang et al. [38] found that patients’ perceptions of the reliability and sincerity of medical staff in service encounters affects their satisfaction with the hospital. Additionally, several studies have attempted to analyze the effects on patient satisfaction by considering the interaction between the patient and medical staff through factors such as communication, responsiveness, and employee reaction [39,40,41]. Here, it should be noted that the quality of care depends primarily on the professional competence of the medical staff [37,38,42]. Therefore, experts need to improve competencies such as knowledge and technical skills to provide high-quality services [42]. However, the ability of the medical staff recognized by the patient is eventually transmitted through the interaction. According to Mosadeghrad [42], quality of care is determined through the cooperation of patients and providers in a supporting environment, which can be achieved when the competencies of the medical staff are premised. Previous studies have shown that if patients form a positive interaction with the medical staff, they trust the medical staff more, which can in turn lead to improved organizational performance. Therefore, the quality of interaction can be considered an important factor in the healthcare context, for which medical service organizations’ efforts to promote long-term relationships are necessary. 

Employee–customer interaction is affected by the servicescape, which represents the surroundings of service encounters. Carù and Cova [43] emphasized that servicescape research should consider the interaction between customers and employees together. In this regard, several previous studies have suggested that although the elements of the servicescape that are important to each encounter are different, the servicescape affects interaction quality in various service settings [3,5]. Additionally, Parish et al. [12], who studied servicescapes in a hospital environment, showed that servicescapes affect employee attitudes. 

### 2.3. Service Quality

Service quality can be defined as the customer’s judgment of the overall excellence or superiority of a service [44]. In recent decades, a great deal of service quality research has been devoted to the development of service quality measures. Among the numerous measurement models, SERVQUAL is an excellent instrument for measuring service quality and is widely applicable in various service industries [45]. According to SERVQUAL, five dimensions are used to assess the perceptions of service quality [44,46]:

- Reliability: ability to perform the promised service accurately;
- Tangibles: service facilities/conditions, equipment, and materials;
- Responsiveness: willingness to help customer and provide service promptly;
- Assurance: knowledge and courtesy of the employee and their ability to inspire confidence;
- Empathy: caring and individualized attention to customers.

Many researchers have conducted research on service quality using SERVQUAL, with several of them identifying limitations and potential difficulties in its application [45,47,48,49,50,51]. Although many problems have been identified in the literature, this study focuses on two main drawbacks of previous studies. The first issue is the problem of conceptualization and the dimensions of SERVQUAL [45,51]. This critique addresses the difficulties in applying and interpreting SERVQUAL [49,52]. The empirical findings of previous studies utilizing SERVQUAL showed a lack of convergent and discriminant validity among the SERVQUAL dimensions [53]. Additionally, the literature acknowledges that an interdimensional overlap may occur [47,49]. To compensate for this problem and analyze how the quality evaluation of services is performed, we focus on process orientation, which is an inherent property of services. A service is a process completed through the customer’s interaction with the service provider. When a service is performed by a person, the dimensions of reliability, assurance, empathy, and responsiveness are ultimately determined by the service provider’s abilities and attitudes [33,54]. Hanks et al. [55] also recognized responsiveness, assurance, and empathy as factors that can be evaluated through interaction and defined them as interpersonal (interaction) quality. Interaction quality can be evaluated according to the emotional exchanges between employees and consumers and the service situation in service encounters [34]. In other words, service encounters based on interpersonal relationships affect service quality significantly [38]. Similarly, Hooper et al. [5] found that employee quality acts as a leading factor in service quality, having a direct and positive effect. Previous studies in the healthcare setting also reported that the quality of service points based on interpersonal relationships affects the overall service quality [38].

Another issue we address are “tangibles”, which refer to physical evidence in service. This dimension of SERVQUAL captures some of the most important aspects of the servicescape [3]. However, it makes it difficult to explain the relationship between dimensions because tangibles are modeled as a factor that measures service quality along with other dimensions. In other words, SERVQUAL does not specify the role of tangibles in the service quality evaluation process. As previously mentioned, servicescapes and tangibles differ. In this study, the servicescape comprehensively identifies and analyzes the elements of the physical environment that the tangible cannot capture, such as ambient conditions, hygiene, and cleanliness. We believe that the servicescape can be a leading factor in overall quality perception by extracting it from SERVQUAL. This inference is based on Mehrabian and Russell [14], who considered that environmental stimuli are useful tools for eliciting customer responses. Specifically, environmental stimuli can elicit emotional states of pleasure and arousal, which ultimately influence behavior. Here, stimuli refer to physical features and can thus be interpreted as a servicescape. Reimer and Kuehn [3] argued that tangibles can capture the tangible parts of a servicescape as a dimension of SERVQUAL and analyzed the causal relationship between servicescapes and other dimensions. Additionally, several previous studies have revealed that the concept of servicescape conflicts with many service quality studies, which include tangible clues as a core dimension, along with various other service quality indicators [3,5,56]. Based on this literature stream, we believe that it is desirable to extract the tangible dimension from SERVQUAL and recognize it as the servicescape. However, the servicescape dealt with in this study is a concept that encompasses not only tangibles, but also the atmosphere and layout. As the servicescape is an immediate element recognized by customers, it is necessary to distinguish it from service quality; therefore, it is necessary to structure it as an antecedent of service quality perception [5].

### 2.4. Behavioral Responses

According to Berry et al. [57], because the customer evaluation of a service is based on performance rather than objectives, customers rely on the numerous clues inherent in performance when evaluating their service experience. Overall, the literature shows that favorable service experiences result in positive behavioral responses, with high levels of service quality [58]. Wakefield and Blodgett [4] argued that in the leisure service industry, consumers’ intentions to revisit increases when the physical environment is well designed. Similarly, Hooper et al. [5] found empirical evidence that the servicescape affects customers’ positive behavioral intentions through retail service research. Regarding interaction quality, Albrecht et al. [59] showed that the experiences from interactions with customers can make customers’ responses more positive. Therefore, we consider the servicescape, interaction quality, and overall quality as clues to the service experience and present their impact on customer responses. Previous studies of the healthcare industry by Sahoo and Ghosh [8] and Choi and Kim [60] have also been conducted under this premise. 

Based on the above discussion, inferences about the relationships between the servicescape, interaction quality, service quality, and patients’ revisit intentions were derived, and the following hypotheses can be established:

**H1:**  *The servicescape has a positive effect on interaction quality*.

**H2:**  *Interaction quality has a positive effect on overall service quality*.

**H3:**  *The servicescape has a positive effect on overall service quality*.

**H4:**  *The servicescape has a positive effect on patients’ revisit intentions*.

**H5:**  *Interaction quality has a significant effect on patients’ revisit intentions*.

**H6:**  *Overall service quality has a significant effect on patients’ revisit intentions*.

## 3. Methodology

### 3.1. Sample, Procedures, and Data Collection

In the Korean medical system, healthcare facilities are divided into medical clinic, hospital, general hospital, and tertiary hospital. A medical clinic is the smallest medical facility with less than 30 beds and mainly treats outpatients with mild symptoms, and also provides routine preventive care. With more than 30 beds, a hospital is a larger facility than a medical clinic and mainly provides medical services for inpatients while performing outpatient treatment like a clinic. General hospitals and tertiary hospitals are large-scale facilities equipped with a full complement of services and departments, typically housing the most experienced and widest range of specialist doctors. Variability in patients’ health status can greatly affect perceptions of environmental factors and interactions that we measure and evaluate. For example, severe or emergency patients who need very serious surgery may be placed in a psychological state in which they are not properly aware of the environment or interactions with medical staff. Therefore, we exclude general hospitals and tertiary hospitals, which are mainly visited by patients suffering from relatively severe diseases, in order to prevent the occurrence of bias due to these external factors in advance. In other words, the sample data we obtained were limited to outpatients who visited clinics and hospitals.

A questionnaire-based survey was administered to patients who received medical treatment within one year at 6 hospitals and 6 medical clinics located in the metropolitan area of Seoul, South Korea. The purpose of the study was explained through an online survey, and a self-administered questionnaire was used for data collection. The survey was conducted over one month, from August to September 2020. We selected 6 clinics and 6 hospitals as target facilities, respectively, and distributed 30 questionnaires each. After removing the discrepancies and incomplete data from the 271 responses collected out of the total of 360, 258 valid responses were used for the final data analysis. In addition, no difference at a 0.05 significance level was found among patient respondents according to hospital/clinic classification. The demographic characteristics of the respondents are summarized in Table 1.

### 3.2. Measures

To ensure content validity, validated measures developed by other scholars were used and adjusted for application in the context of healthcare services. Table 2 shows the operational definitions of the constructs and the primary sources of the instruments. The measurement items for the servicescape were adapted from Wakefield and Blodgett [61], Hightower et al. [56], Reimer and Kuehn [3], and Hooper et al. [5]. A total of 15 items were used to measure the five dimensions of the servicescape: equipment, design, space, ambience, and hygiene. Interaction quality was assessed using five items, adapted from Stevens et al. [54] and Chang et al. [38]. Items relating to service quality were adapted from Grace and O’Cass [62] and Hooper et al. [5], whereas those relating to revisit intention were drawn from Zeithaml et al. [58]. The final measurement items for each construct are based on a five-point Likert-type scale, from “1” for “strongly disagree” to “5” for “strongly agree”. As this survey was conducted in Korea, it was necessary to translate the text into Korean. The original version of the questionnaire was translated into Korean by the corresponding author and then back translated into English by another bilingual scholar. Next, we proceeded with an item-by-item review, and the corresponding author confirmed that there was no difference between the two versions. The items for all constructs are presented in Appendix A. 

### 3.3. Data Analysis and Results

Survey data were analyzed using statistical techniques, including confirmatory factor analysis (CFA) and structural equation modeling. Prior to testing the hypotheses, we examined the validity and reliability of the two-step approach [63,64]. CFA was performed using AMOS 21 software to assess the factor loadings, internal consistency, and convergent validity of all measurement scales. Factor loadings and Cronbach alphas greater than 0.7 are considered acceptable [65]. All items in this study were retained, except for SP1, AM3, and AM4, which were removed due to low loadings. Composite reliability (CR) values were above the recommended value of 0.7 and all item loadings for each reflective construct were greater than 0.5. Additionally, there was no problem with convergent validity in that the average variance extracted (AVE) of all constructs exceeded 0.5. Table 3 reports the factor loadings, AVE, CR, and Cronbach alphas. According to Fornell and Larcker [65], when the square root of all AVEs exceeds all cross-correlation scores, there is no problem with discriminant validity. As shown in Table 4, most of the square roots of AVE were higher than all cross-correlation scores. Although the interaction and service quality did not meet this criterion, the corresponding cross-correlation scores were not extremely high and their CR values were very high, at 0.873 and 0.911, respectively; thus, discriminant validity does not cause a serious problem [66]. Altogether, the results indicate that all constructs are statistically distinct and can be used to test the structural model.

The model fit statistics indicate a good fit for the final measurement model and are well within the recommended thresholds: χ2/df = 1.999 (χ2 = 495.98, df = 248), RMSEA = 0.062, GFI = 0.686, CFI = 0.943, and IFI = 0.944. Path coefficients were used to test the hypotheses. H2, H5, and H6 were supported, while H3 and H4 were not. H1 was partially supported. First, for H1, space has a strong positive effect on interaction quality (β = 0.193, *t* = 2.760, *p* < 0.01). Hygiene also has a strong positive effect on interaction quality (β = 0.658, *t* = 7.109, *p* < 0.001). However, H1(a), H1(b), and H1(d) were not statistically supported as hypotheses related to equipment, design, and atmosphere, which are other components of the servicescape. The direct path from interaction quality to service quality (β = 0.875, *t* = 7.671, *p* < 0.001) thus supported H2. All paths from the servicescape to service quality and revisit intention were insignificant, thus leading to the rejection of H3 and H4. The results also indicate that H5 and H6 were supported by positive and significant paths from interaction quality (β = 0.531, *t* = 2.362, *p* < 0.05) and service quality (β = 0.450, *t* = 3.296, *p* < 0.001) to revisit intention. The results of the hypotheses testing are presented in Table 5.

We used a bootstrapping approach with phantom variables to investigate the significance of mediating effects. According to Malhotra et al. [67], this method is preferred because it is statistically more powerful and robust than the methods of Baron and Kenny [68] and Sobel [69] for identifying mediation effects. In the case of analyzing multiple mediation in a model, the bootstrapping of individual indirect effects must be performed, so phantom variables were created and each indirect effect was expressed as a single coefficient for analysis [70]. Specifically, we used a bootstrap analysis of 2000 resamples, from which a bias-corrected 95% confidence interval was estimated. Mediating effect analysis focuses on the relationships in which interaction quality and service quality act as mediators in a proposed research model. First, we presented the mediating effects of interaction quality on the relationship between the servicescape and service quality. The results demonstrate that the impact of space and hygiene on service quality is significantly mediated by interaction quality. H3, the hypothesis that the servicescape has a direct and positive effect on service quality, was rejected; this result is very interesting. The mediating effect of interaction quality on revisit intention showed similar results. In other words, interaction quality has a significant mediating effect on the relationship between space, hygiene, and revisit intention. Second, we analyzed the mediating effects of service quality. In other words, this verifies whether service quality has a mediating effect on the relationship between interaction quality and revisit intention. The results confirmed that service quality significantly mediates the influence of interaction quality on revisit intention. Additionally, hygiene is statistically significant, as it has an indirect effect by mediating interaction between quality and service quality. The results of the mediating analysis using the estimates of the direct, indirect, and total effects are shown in Table 6. 

## 4. Discussion

### 4.1. Findings and Managerial Implications

The space and hygiene of servicescapes were found to have substantial effects on interaction quality. Hooper et al. [5] used the same constructs for servicescapes as this study and reported that all factors of a servicescape significantly affect interaction quality, which is somewhat different from the results of this study. The importance of servicescapes may differ, depending on industry characteristics. Unlike contact employees in other service industries, medical staff must treat patients who visit facilities because of health concerns. In other words, the servicescape required by the medical staff can be the spatial element to provide services quickly and safely. Additionally, the importance of hygiene is revealed as an environmental factor that is directly related to health, which provides the essential value of healthcare services. This finding suggests that medical staff can elicit a high level of interaction with patients through an environment that can appropriately support rapid treatment or examination of medical conditions, rather than building design, ambience, and state-of-the-art equipment.

Interaction quality was found to have a significant influence on service quality. This result is in agreement with that of a previous study on service quality in a healthcare setting [38]. However, the direct effect of servicescapes on service quality has not yet been verified. Accordingly, we explored the indirect effect of servicescapes on service quality through a mediating effect analysis and drew interesting findings. Space and hygiene were found to have significant indirect effects on service quality. This result was confirmed by isolating interaction-related factors to explain the overall service perception. Specifically, we presented empirical evidence that space and hygiene not only directly affect interaction quality, as described above, but also affect the overall service quality perceived by patients through interaction quality. Therefore, the purpose of this study, to analyze how the servicescape can affect employees and customers from multiple angles, has been achieved. This finding suggests that healthcare organizations such as hospitals and clinics should not simply use a straightforward approach when managing servicescapes. In other words, maintaining a high level of hygiene and space may not directly affect customers’ perceptions of quality and behavior. The results indicate that factors such as hygiene and space also provide an environment in which employees can perform their jobs well. Although it is true that servicescapes affect everyone who encounters service surroundings, it is also undeniable that they have been mainly treated as a factor for customers, both theoretically and practically. This study emphasizes that hygiene and space management can encourage medical staff. This ultimately allows patients to feel satisfied and return. 

The hypothesis test on revisit intention failed to test the direct positive influence of the servicescape. That is, the servicescape did not directly affect service quality or the intention to revisit. However, revisit intention was significantly affected by both interaction and service quality. Therefore, we analyzed whether the servicescape affects revisit intention by mediating service quality, finding that hygiene and space indirectly affect revisit intention. In particular, the effects of hygiene were stronger. Furthermore, hygiene was found to be the only servicescape factor affecting revisit intention through double mediation. This implies that hygiene is the most important servicescape factor in terms of patient revisits for healthcare services. Space is the second servicescape that can influence patient revisits. This further reinforces the evidence of the relationship between the servicescape and service quality. Since the space and hygiene of servicescapes indirectly affect not only service quality but also revisit intention, healthcare organizations should make efforts to foster space and hygiene aspects in making decisions about the facility environment. Additionally, interaction quality was found to have a significant effect on revisit intention, although not as much as the outcome of the service. This result implies that staff responses and attitudes toward patients can directly affect patients’ revisit intentions. These results suggest that in a service industry where human capabilities are important, such as medical services, interactions between customers and service employees can directly lead to customer revisits. In particular, it shows that the attitudes and behaviors of medical staff can help patients stabilize in healthcare facilities where patients visit in a state of psychological instability. In South Korea, where this study was conducted, there is already a system in place that allows all medical organizations’ staff to acquire the basic medical service mindset and service response knowledge necessary for patient response in the medical field.

### 4.2. Theoretical Contributions

This study contributes to the literature in two ways, as follows. The most significant theoretical contribution is the development of a framework for analyzing customer perceptions and behaviors. Many studies have emphasized that the physical environment of service facilities and the interactions between employees and customers are important factors in the overall perceptions of customers during service encounters. However, while attention has been paid to expanding the typologies of service quality or the servicescape, relatively little effort has been made to identify the relationships between the service elements perceived by customers while experiencing a service [38,42]. For example, SERVQUAL, one of the most widely accepted models for evaluating service quality, includes physical elements, such as tangibles, and interaction elements, such as responsiveness, assurance, and empathy. Moreover, the recently reported extended servicescape model includes interpersonal aspects, such as considering social factors in addition to the physical environment [16,17,71]. Therefore, it is necessary to propose a model that can structurally analyze environmental and interpersonal aspects, which cannot be identified in the extant theoretical frameworks, along with overall quality perception. This study contributes to the literature by proposing and empirically verifying the relationship between servicescapes, interaction quality, and overall service quality by reviewing previous studies of the service elements related to these aspects. Additionally, the above-mentioned structural model has been expanded by identifying the customer behavior the service organization places importance on as the patient’s intention to revisit. 

The second contribution is specific to healthcare services. The research model was designed in consideration of the servicescape as an antecedent to patients’ perceptions and behavioral intentions, thus enabling the analysis of various relationships. However, the healthcare literature did not provide unified results in analyzing the effect of servicescapes on patient emotions or satisfaction. For example, Ayas et al. [72] argued that space, design, and ambience are important for a patient’s feeling of calmness, while Sahoo and Ghosh [8] reported that design and atmosphere are the main factors influencing patient satisfaction, and hygiene is not. Further, Pai and Chary [20] argued that hygiene is the most important factor, as opposed to design, space, and amenities. This study confirms that hygiene is the most influential factor among the servicescape dimensions. The implication is that, among the environmental factors of healthcare facilities, hygiene and space become direct driving forces for a smooth interaction with patients and can be strategic tools to promote service quality and patient revisits. Of course, it cannot be concluded that there are no patients who return to unsanitary facilities. In particular, there may be cases where a visit is unavoidable due to geographical proximity or because of the superior expertise of medical personnel, but these cases are considered exceptional or special ones. However, in a service area where expertise is important, such as medical service, a study on whether service expertise can dominate environmental factors needs to be conducted later. Securing the professionalism of medical staff is undoubtedly important in promoting the competitiveness of medical institutions. However, it is also important to discuss how to effectively utilize human resources. In other words, it is worth researching how to improve the quality of the interactions between the medical staff and patients. This study provides a framework for analyzing the relationships between environmental factors and the interactions between staff and patients. However, the roles of hygiene and space identified in this study may be due to the restrictions imposed by the COVID-19 pandemic. The pandemic has placed a tremendous psychological burden on both medical staff and patients. Accordingly, there is a possibility that when patients perceive the surroundings during service encounters, the hygiene and space aspects are more important than the other servicescape factors. Of course, as this may be a phenomenon that can be commonly applied not only to healthcare services but also to other service areas, further research is needed to verify whether these results are caused by the spread of infectious diseases. If a patient’s perception of the environment of healthcare services changes due to frequent epidemics, such as SARS, MERS, and COVID-19, additional research is needed to determine whether the results will be sustained.

### 4.3. Limitations and Future Research

Although this study makes significant contributions to the service and healthcare management literature and has important implications for practice, it also has several limitations and provides opportunities for future research. First, the data used in this study were collected during the COVID-19 pandemic, meaning that we need to pay attention to the phenomenon of selective attention. Focusing on one stimulus may have led to selective attention and actively ignoring the other stimuli. In other words, patients may be insensitive to other environmental stimuli because they pay much attention to prevention, owing to the spread of an infectious disease. For this reason, the effects of design, ambience, and equipment may be insignificant, which makes it the researcher’s responsibility to confirm the changes after the pandemic through further analysis. Second, the data include the perceptions of patients who have experienced healthcare services, including both hospitals and clinics. Therefore, there is a possibility that the servicescape effect may be somewhat distorted. Since the environmental factors for inpatient and outpatient values may differ, future studies will be able to enrich practical implications by classifying them and analyzing the differences. Finally, in the healthcare context, we have developed a broad picture of the servicescape, quality perception, and patient behavior. However, these relationships may not be the same in all medical fields (departments). For example, the effects shown in this study may be greater in otolaryngology than in orthopedics. Future research should thus investigate the effects of these characteristics in the general healthcare field. Another interesting research topic is the interpersonal aspects between customers. As mentioned in the literature review, social factors include not only the interactions between employees and customers but also those between customers. Instead of excluding the interactions between patients, our study focuses on interactions between customers and patients. Therefore, future research using the framework proposed in this study will also be conducted in hospitals that accommodate inpatients.

## 5. Conclusions

This study developed a conceptual framework that encompasses the servicescape and patients’ perceptions and behaviors, and conducted an empirical investigation of healthcare service facilities. With the recent spread of viruses such as COVID-19, social awareness of hygiene is increasing, and healthcare facilities in particular are managing the physical environment to a higher level than before. Along with these environmental factors, the intention was to analyze how the expertise of medical services and the interaction provided by medical staff ultimately affect the overall quality of medical facilities. Furthermore, it was analyzed in depth whether these factors were motivating patients to visit the facilities again. Through empirical investigation, it was found that service quality can be improved and patients’ revisits to the facilities can be induced through servicescape improvement and interaction quality.

## Figures and Tables

**Table 1:** Demographic characteristics of respondents.

Classification	Frequency	Percentage
**Gender**		
Male	123	47.7
Female	135	52.3
**Age**		
20s	56	21.7
30s	74	28.7
40s	89	34.5
50s	39	15.1
**Marital status**		
Single	122	47.3
Married	133	51.6
Others	3	1.2
**Education**		
High school diploma	56	21.7
Associate degree	27	10.5
Bachelor’s degree	145	56.2
Master’s degree/PhD	30	11.6
**Monthly income**		
KRW 2,000,000 or less	68	26.4
KRW 2,000,000–3,000,000	65	25.2
KRW 3,000,000–4,000,000	52	20.2
KRW 4,000,000–5,000,000	28	10.9
KRW 5,000,000 or more	45	17.4
**Reason for visiting the hospital/clinic**		
Treatment	177	68.6
Beauty and wellness	16	6.2
Prevention	55	21.3
Others	10	3.9

Notes: All currency values are in KRW (Korean Republic Won); KRW 1188 = USD 1 (date: 20 August 2020).

**Table 2:** Construct operationalization.

Construct	Definition	Primary Sources
**Servicescape**		
Equipment	Modern configuration, ease, and convenience of equipment or devices	[61]
Design	Attractiveness of design elements found in the exterior and interior of the facility	[3,5]
Space	Safety of the floor and space and the marking of the movement route	[5,61]
Ambience	Proper control of indoor background music, illuminance, and smell	[3,56]
Hygiene	Cleanliness and hygiene to prevent infection	[3,5]
**Interaction quality**	Staff’s ability to respond, including kindness, communication, work ability, and attitude toward patients	[38,54]
**Service quality**	Overall service evaluation of consumers using hospitals and clinics	[5,62]
**Revisit intention**	Patient’s intention to visit continuously, whether to recommend hospitals or clinics to patients with the same disease, and the positive word-of-mouth effect	[58]

**Table 3:** Factor loading, average variance extracted, composite reliability, and Cronbach alpha.

Construct	Item	Loading	*t* Value	AVE	CR	Cronbach α
Equipment	EQ1	0.658		0.582	0.805	0.73
	EQ2	0.673	8.669 ***			
	EQ3	0.748	9.297 ***			
Design	DS1	0.778	-	0.689	0.869	0.843
	DS2	0.856	13.698 ***			
	DS3	0.778	12.560 ***			
Space	SP1	Eliminated				
	SP2	0.52	-	0.574	0.574	-
Ambience	AM1	0.993	-	0.843	0.913	0.882
	AM2	0.795	13.122 ***			
	AM3	Eliminated				
	AM4	Eliminated				
Hygiene	HC1	0.798	-	0.731	0.890	0.838
	HC2	0.820	13.971 ***			
	HC3	0.778	13.142 ***			
Interaction Quality	IQ1	0.786	-	0.674	0.911	0.873
	IQ2	0.758	13.040 ***			
	IQ3	0.751	12.897 ***			
	IQ4	0.774	13.387 ***			
	IQ5	0.744	12.740 ***			
Service Quality	SQ1	0.848	-	0.757	0.939	0.911
	SQ2	0.842	17.166 ***			
	SQ3	0.814	16.233 ***			
	SQ4	0.776	15.038 ***			
	SQ5	0.825	16.596 ***			
Revisit Intention	RI1	0.817	-	0.783	0.915	0.902
	RI2	0.885	17.070 ***			
	RI3	0.906	17.610 ***			

Notes: *** *p* < 0.001; CR: composite reliability; AVE: average variance extracted.

**Table 4:** Square root of AVE and correlations between constructs.

	EQ	DS	SP	AM	HC	IQ	SQ	RI
EQ	0.758							
DS	0.445	0.763						
SP	0.523	0.725	0.830					
AM	0.502	0.563	0.588	0.918				
HC	0.549	0.690	0.660	0.533	0.855			
IQ	0.577	0.601	0.543	0.489	0.773	0.821		
SQ	0.595	0.655	0.581	0.510	0.837	0.915	0.870	
RI	0.530	0.578	0.513	0.422	0.645	0.844	0.829	0.885

Notes: The diagonal elements indicate the square root of the AVE. AVE measures the amount of variance captured by the measures of a construct in relation to error variance of those items.

**Table 5:** Results of the structural model.

Paths		Path Coefficients	*t* Value	Results
Equipment → Interaction Quality	H1	0.162	1.617	Not supported
Design → Interaction Quality		−0.089	−0.996	Not supported
Space → Interaction Quality		0.193	2.760 **	Supported
Ambience → Interaction Quality		0.018	0.298	Not supported
Hygiene and Cleanness → Interaction Quality		0.658	7.109 ***	Supported
Interaction Quality → Service Quality	H2	0.875	7.671 ***	Supported
Equipment → Service Quality	H3	−0.011	−0.116	Not supported
Design → Service Quality		−0.003	−0.037	Not supported
Space → Service Quality		0.044	0.659	Not supported
Ambience → Service Quality		0.020	0.369	Not supported
Hygiene and Cleanness → Service Quality		0.015	0.139	Not supported
Equipment → Revisit Intention	H4	0.100	1.033	Not supported
Design → Revisit Intention		0.063	0.728	Not supported
Space → Revisit Intention		0.039	0.551	Not supported
Ambience → Revisit Intention		−0.047	−0.834	Not supported
Hygiene and Cleanness → Revisit Intention		−0.249	−2.114	Not supported
Interaction Quality → Revisit Intention	H5	0.531	2.362 *	Supported
Service Quality → Revisit Intention	H6	0.450	3.296 ***	Supported

Notes: * *p* < 0.05, ** *p* < 0.01, *** *p* < 0.001.

**Table 6:** Direct, indirect, and total effects.

Path	Direct Effect	Indirect Effect	Total Effect
Equipment → Service Quality	−0.011	0.142	0.131
Design → Service Quality	−0.003	−0.078	−0.081
Space → Service Quality	0.044	0.169 *	0.213
Ambience → Service Quality	0.020	0.016	0.036
Hygiene → Service Quality	0.015	0.585 **	0.600
Equipment → Revisit Intention	0.100	0.142	0.242
Design → Revisit Intention	0.063	−0.083	−0.020
Space → Revisit Intention	0.039	0.200 *	0.239
Ambience → Revisit Intention	−0.047	0.027	−0.020
Hygiene → Revisit Intention	−0.249	0.619 **	0.370
Interaction Quality → Revisit Intention	0.450	0.465 *	0.915
Space → Interaction Quality → Revisit Intention	-	0.085	-
Space → Service Quality → Revisit Intention	-	0.023	-
Space → Interaction Quality/Service Quality → Revisit Intention	-	0.088	-
Hygiene → Interaction Quality → Revisit Intention	-	0.323	-
Hygiene → Service Quality → Revisit Intention	-	0.009	-
Hygiene → Interaction Quality/Service Quality → Revisit Intention	-	0.334 *	-

Note: * *p* < 0.05, ** *p* < 0.01.

## Data Availability

The data presented in this study are available on request from the corresponding author. The data are not publicly available due to ethical issues.

## Appendix A. Measurement Items

**Item**	**Notation**	**Description**
Servicescape		
Equipment	EQ1	The equipment was modern looking.
	EQ2	The electronic equipment was excellent.
	EQ3	The equipment was of high quality.
Design	DS1	The architecture was attractive.
	DS2	I found the interior design visually appealing.
	DS3	The color schemes were appropriate.
Space	SP1	The flooring was appropriate.
	SP2	I found my way around easily.
Ambience	AM1	The background music was pleasant.
	AM2	The background music was appropriate.
	AM3	The lighting was comfortable.
	AM4	The hospital/clinic had a pleasant smell.
Hygiene	HC1	The service station appeared to be hygienic and excellent in preventing infection.
	HC2	The hospital/clinic was very clean.
	HC3	The medical staff were neat and tidy in appearance.
Interaction quality	IQ1	Personnel possessed the required know-how needed to effectively deliver the treatment.
	IQ2	Providing services that really earn patients confidence in the service encounter.
	IQ3	Providing services that instantly and rapidly respond to patients’ demands.
	IQ4	Providing services that correctly delivery the service requested by patients.
	IQ5	Providing services that make you feel comfortable and confident.
Service quality	SQ1	The hospital/clinic provided good service.
	SQ2	The service suited my needs.
	SQ3	The service was reliable.
	SQ4	The service station provided quality service.
	SQ5	The service was of a very high quality.
Revisit intention	RI1	I am willing to visit this hospital/clinic continuously.
	RI2	I will recommend hospital/clinic for patients with the same symptoms.
	RI3	I will tell my family and close acquaintances positive stories about this hospital/clinic.
